# Increased Prevalence of Myopia in the United States between 1971 and 1972 and 1999 and 2004—A Reappraisal

**DOI:** 10.1016/j.xops.2025.100786

**Published:** 2025-04-04

**Authors:** Mark A. Bullimore, Xu Cheng, Noel A. Brennan

**Affiliations:** Houston, Texas; Jacksonville, Florida; Melbourne, Australia

The prevalence of myopia in the United States population was estimated to increase from 25% to 42% over 30 years between the 1971–1972 and 1999–2004 National Health and Nutrition Examination Survey.^1^ Nonetheless, there were unexplained anomalies in the data. For example, those aged 18 to 24 years in 1971–1972 (who would be 45–57 years of age in 1999–2004) had myopia prevalence of 28%, but those aged 45 to 54 years in 1999–2004 had myopia prevalence of 45%, suggesting meaningful adult-onset myopia. Here, we reappraise these data and propose a model to account for the 30-year age-dependent changes in prevalence.

We developed a theoretical framework of myopia prevalence by age and time, allowing for both increased prevalence and adult myopia onset and progression between surveys.

**Figure 1:**
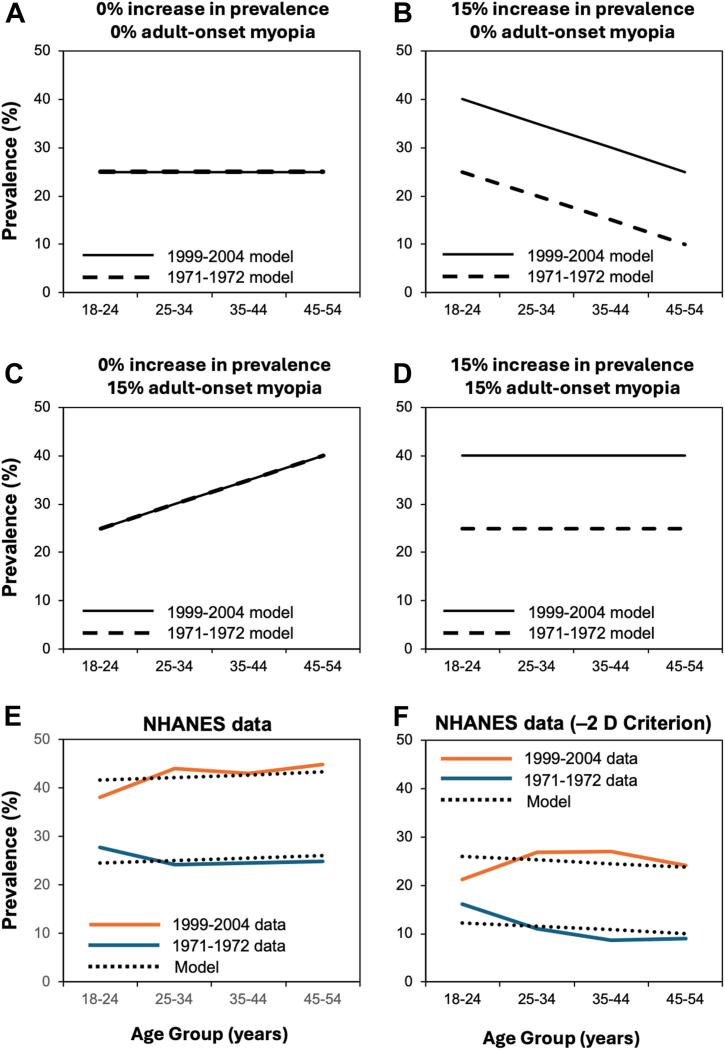
Prevalence of myopia. **A,** No increased prevalence over time or adult-onset myopia. **B,** A 15% increase in prevalence, but no adult-onset myopia. **C,** No increased prevalence over time but 15% adult-onset myopia. **D,** Both a 15% increase in myopia prevalence and 15% adult-onset myopia. **E,** NHANES myopia prevalence data modeled by an increase of 13.0% in prevalence and an increase of 17.1% due to adult-onset myopia. **F,** NHANES myopia prevalence data and modeled by an increase of 8.8% in overall population prevalence for this threshold accompanied by an increase of 13.7% due to adult myopic progression. NHANES = National Health and Nutrition Examination Survey.

Data from the 12- to 17-year age group were not considered as they would be most susceptible to the lack of cycloplegia. By way of illustration, Figure 1A assumes a starting prevalence of 25% among those aged 18 to 24 years in 1971–1972, with no increased prevalence or adult-onset myopia over time. In Figure 1B, an increase of 15% prevalence between surveys is assumed without any adult progression. Figure 1C shows adult-onset myopia of 15% in the absence of change in prevalence over time. Finally, in Figure 1D, both an overall increase of myopia prevalence of 15% and adult onset of 15% are modeled.

An unweighted linear regression was performed to model the influence of sex, change in prevalence across time, and adult-onset myopia using data from of Vitale et al.^1^ A similar process was performed for a threshold of −2 diopters (D) (their Table 1), with the assumption that any changes in prevalence would be due to adult myopia progression, rather than onset.

There was a significant interaction whereby greater myopia prevalence among women at a younger age disappeared in the older age group. This observation aside, myopia prevalence data are best explained by an increase of 17.1% in overall population prevalence—consistent with Vitale et al^1^—and with 18.9% due to adult-onset myopia (Fig 1E).

For a myopia threshold of −2 D (Fig 1F), an increase of 13.7% in overall population prevalence was accompanied by 11.4% adult myopic progression.

The differences between the National Health and Nutrition Examination Survey data from 1971–1972 to 1999–2004 are best explained by not only an increase in overall population myopia prevalence, but a substantial component of adult-onset myopia and progression. A recent comprehensive review concluded that adult-onset may represent a third or more of all myopia in western populations, but less in East Asia, where onset during childhood is high.^2^ The model presented here supports that conclusion with 19% of all individuals developing myopia beyond the age of 18 years. The review also indicates that clinically meaningful myopia progression continues in early adulthood that may average −1 D between the ages of 20 and 30 years. This latter assertion is supported by our recent analysis of 3 studies, including the 1971–1972 and 1999–2004 National Health and Nutrition Examination Survey data,^1^^,^^3^^,^^4^ that all provide evidence of around −1 D myopia progression between the ages of 20 and 50 years.^5^

We have used a simple analysis of summary data here and more sophisticated modeling using source data might be more informative. For example, a nonsignificant trend for greater adult-onset myopia in the 1999–2004 survey in our analysis could be investigated. Furthermore, most adult-onset myopia occurs before 30 years of age; however, our regression assumes a constant incidence rate with age. Nonetheless, our analysis suggests that interpreting the increasing prevalence over 30 years is informed by both substantial adult-onset myopia and adult myopia progression.
